# Supplementary material for: Predictors of Repeat Medical Emergency Team Activation in Deteriorating Ward Patients: A Retrospective Cohort Study
Source: J Clin Med. 2022 Mar 21;11(6):1736. doi: 10.3390/jcm11061736 (PMC8950705; doi:10.3390/jcm11061736)
Supplement: Supplementary file 1 [file jcm-11-01736-s001.zip › jcm-1604561-supplementary.pdf]

Supplementary Materials

Table S1. Subgroup analysis in patients with medical emergency team activation due to respiratory causes.

Variables	Repeat MET Activation		*p*
	**Yes (*n* = 531)**	**No (*n* = 2162)**	
Age, yr	66 (53–74)	66 (55–74)	0.704
Male gender	358 (67.4)	1378 (63.7)	0.117
Department			0.004
Medicine	490 (92.3)	1899 (87.8)	
Surgery	41 (7.7)	263 (12.2)	
Comorbidities			
Solid tumor	156 (29.4)	1215 (56.2)	<0.001
Hematological malignancies	121 (22.8)	213 (9.9)	<0.001
Chronic lung disease	120 (22.6)	381 (17.6)	0.011
Chronic heart disease	256 (48.2)	970 (44.9)	0.173
Chronic liver disease	42 (7.9)	240 (11.1)	0.033
Chronic renal disease	29 (5.5)	91 (4.2)	0.240
Vital parameter at first MET activation			
Systolic blood pressure (mmHg)	123 (107–140)	124 (109–142)	0.007
Heart rate (beats/min)	110 (93–126)	109 (93–123)	0.278
Respiratory rate (breaths/min)	27 (22–32)	26 (22–31)	0.001
Body temperature (℃)	36.9 (36.5–37.6)	36.8 (36.5–37.4)	0.124
SpO_2 (%)	94 (90–97)	93 (89–97)	0.186
FiO_2 (%)	0.50 (0.32–0.55)	0.45 (0.29–0.50)	<0.001
SpO_2/FiO_2 ratio	200 (140–229)	236 (182–350)	<0.001
Modified early warning score	5 (3–6)	4 (3–6)	<0.001
SOFA	6 (4–8)	4 (3–6)	<0.001

MET = medical emergency team; SOFA = sequential organ failure assessment. Data are expressed as median with interquartile range or number (percentage).
